# Combined resection of the gastroduodenal artery without revascularization in distal pancreatectomy with *en bloc* celiac axis resection (extended DP-CAR) for pancreatic cancer: A case report

**DOI:** 10.1016/j.ijscr.2022.107803

**Published:** 2022-11-29

**Authors:** Atsushi Tomioka, Mitsuhiro Asakuma, Nao Kawaguchi, Koji Komeda, Tetsunosuke Shimizu, Sang-Woong Lee

**Affiliations:** General and Gastroenterological Surgery, Osaka Medical and Pharmaceutical University

**Keywords:** CA, celiac axis, CHA, common hepatic artery, CT, computed tomography, DP-CAR, distal pancreatectomy with *en bloc* celiac axis resection, GDA, gastroduodenal artery, IMV, inferior mesenteric vein, ISGPF, International Study Group of Pancreatic Fistula, LAPC, locally advanced pancreatic cancer, LGA, left gastric artery, NCCN, The National Comprehensive Cancer Network, PHA, proper hepatic artery, PV, portal vein, r-RHA, replaced right hepatic artery, r-LHA, replaced left hepatic artery, SMA, superior mesenteric artery, SMV, superior mesenteric vein, SpA, splenic artery, UICC, The Union for International Cancer Control, Case report, Extended DP-CAR, DP-CAR, Locally advanced pancreatic cancer, Gastroduodenal artery

## Abstract

**Introduction:**

Distal pancreatectomy with *en bloc* celiac axis resection (DP-CAR) is performed to remove locally advanced pancreatic cancer (LAPC) that involves the celiac axis (CA), the common hepatic artery (CHA), or the root of the splenic artery (SpA). It is not usually applied to LAPC involving both the CA and the gastroduodenal artery (GDA) because transection of the GDA cannot assure hepatic perfusion. Preserving the replaced hepatic artery might allow combined resection of the GDA without revascularization.

**Presentation of case:**

A 78-year-old woman who was diagnosed with LAPC of the pancreatic head and body that invaded the GDA and proper hepatic artery, as well as the CA. The left hepatic artery (LHA) was solitarily branched from the left gastric artery (LGA), which was branched from proximal to the confluence of the CHA and the SpA. The root of the LGA was intact. We successfully performed DP-CAR with combined resection of the GDA, without revascularization, by preserving the LGA.

**Discussion:**

This is the first English literature case of extended DP-CAR with preservation of the replaced LHA (r-LHA). Aberrant right and left hepatic arteries are common variations. Checking the arterial variations is very important when deciding the treatment strategy for LAPC, especially in cases that appear unresectable.

**Conclusion:**

Our case indicated that the r-LHA alone can supply the entire liver in extended DP-CAR. The resectability must be decided with close evaluations of the vessel variations and the tumor status.

## Highlights

• Locally advanced pancreatic cancer involving celiac axis and gastroduodenal artery
• DP-CAR with combined resection of gastroduodenal artery without revascularization
• First English literature case of extended DP-CAR with replaced LHA preserved
• Significance of arterial variations when deciding the treatment strategy

## 1 Introduction

Distal pancreatectomy with *en bloc* celiac axis resection (DP-CAR) is a surgical procedure for removing locally advanced pancreatic cancer that involves the celiac axis (CA), the common hepatic artery (CHA), or the root of the splenic artery (SpA) [1]. In this procedure, after celiac resection and ligation of the distal CHA, perfusion of the liver relies on only pancreaticoduodenal arcades to the gastroduodenal artery (GDA) and proper hepatic artery (PHA) via the superior mesenteric artery (SMA). Because transection of the GDA cannot assure hepatic perfusion, DP-CAR is not usually applied to locally advanced pancreatic cancers involving both the CA and the GDA. Combined resection of GDA and PHA, as well as reconstruction, is technically possible; however, it is associated with an increased risk of perioperative mortality [2]. Therefore, arterial reconstruction in pancreatic resection is performed only in limited facilities and is not commonly used. However, if there is an aberrant hepatic artery, preserving it may safely and effectively allow combined resection of GDA or PHA in DP-CAR without revascularization. We herein report a case of locally advanced pancreatic cancer treated by DP-CAR with preservation of the replaced left hepatic artery (r-LHA) and combined resection of the GDA without revascularization. This work has been reported in line with the SCARE 2020 criteria [3].

## 2 Presentation of case

**Fig. 1:**
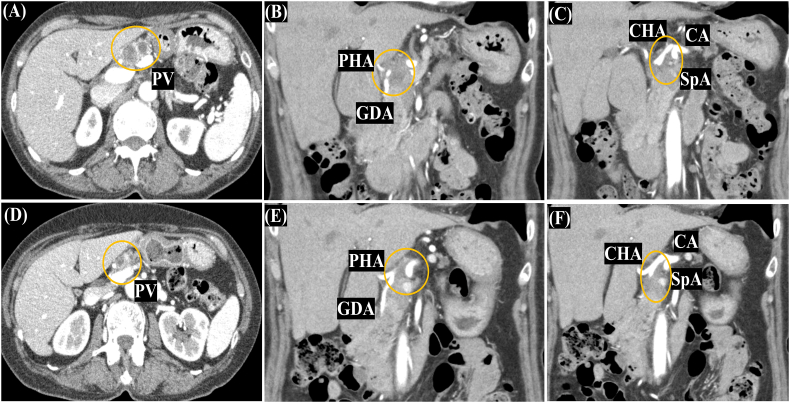
Abdominal CT: Pre-chemotherapy (A, B, C), post-chemotherapy (D, E, F). Computed tomography (CT) demonstrated a low attenuation solid mass on the PV/SMV (A, D) that is invading the GDA (B, E) and has an abutment with CA (C, F).

A 78-year-old woman who was checked regularly by chest computed tomography (CT) because she had a resection of a thymoma and radiation therapy 29 years ago. She was also taking oral prednisolone. The last CT chest detected a pancreatic tumor and dilatation of the main pancreatic duct by chance. She was asymptomatic. Contrast abdominal CT showed a low attenuated tumor at the pancreatic head and body invading the GDA and PHA. Furthermore, the tumor had extended along the CHA beyond the root of the SpA and the abutment with the CA was recognized (Fig. 1). EUS-FNA pathologically diagnosed the tumor as an adenocarcinoma. There was no evidence of distant metastasis or lymph node metastasis. Physiological and laboratory examinations, including tumor markers, were unremarkable as follows: CA19–9 = 31.7 U/mL, CEA = 4.1 ng/mL, DUPAN-2 = 25 U/mL, and Span-1 = 16 U/mL. The diagnosis was locally advanced pancreatic ductal adenocarcinoma (cT4, cN0, cM0, cStage III UICC 7th).

**Fig. 2:**
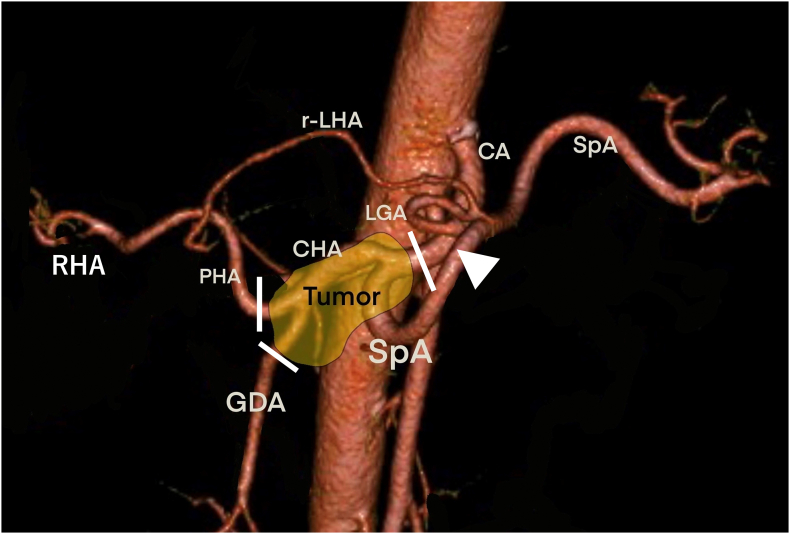
3D-CT angiography. The 3D-CT angiography reveals the existence of r-LHA. The root of the LGA (arrowhead) was not invaded by the tumor. The white lines show transected points of arteries.

We performed systemic chemotherapy consisting of gemcitabine 1000 mg/m^2^ and nab-paclitaxel 125 mg/m^2^ on days one, eight, and 15 monthly. After six months, the tumor size was stable and was still in contact with the CHA, SpA, GDA, and PHA. The tumor marker levels were still within the normal range. The arterial phase of the contrast abdominal CT revealed that the left hepatic artery was solitarily branched from the LGA, which was branched from proximal to the confluence of the CHA and the SpA. The root of the LGA was not invaded by the tumor (Fig. 2).

This case involves a distant pancreatectomy with CA resection, and combined resection of the GDA and PHA resection to completely remove the tumor.

If it were not for the aberrant hepatic artery, revascularization of the proper hepatic artery would be inevitable and radical surgery would be inapplicable. However, we believed that preserving the LGA would allow adequate perfusion to the entire liver via the r-LHA.

**Fig. 3:**
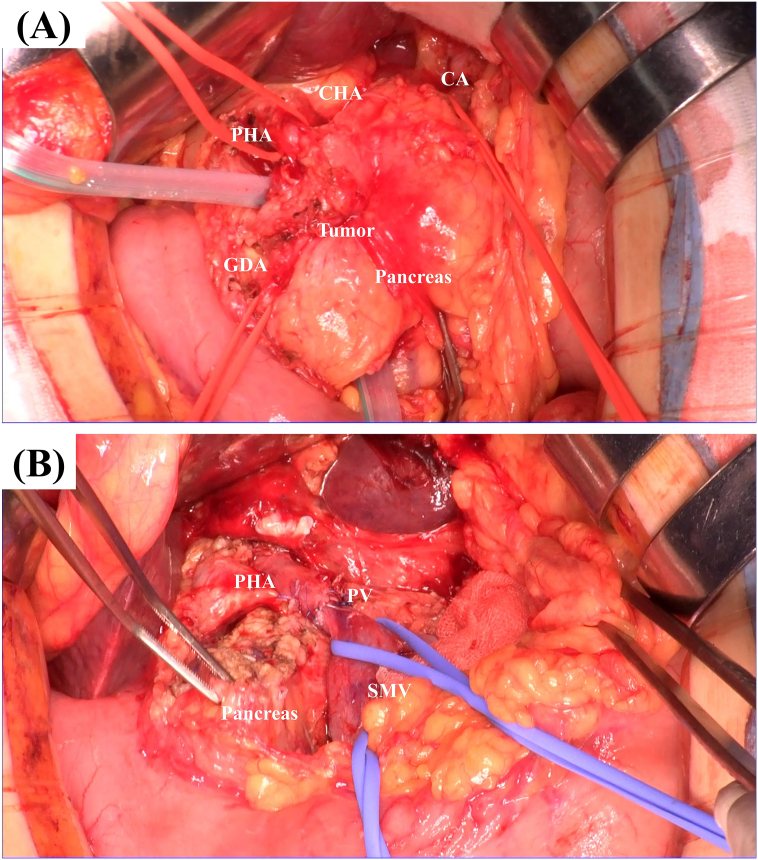
Views in the operation. The GDA and PHA were involved in the tumor (A). Extended DP-CAR was performed. The PV and SMV were reconstructed (B).

**Fig. 4:**
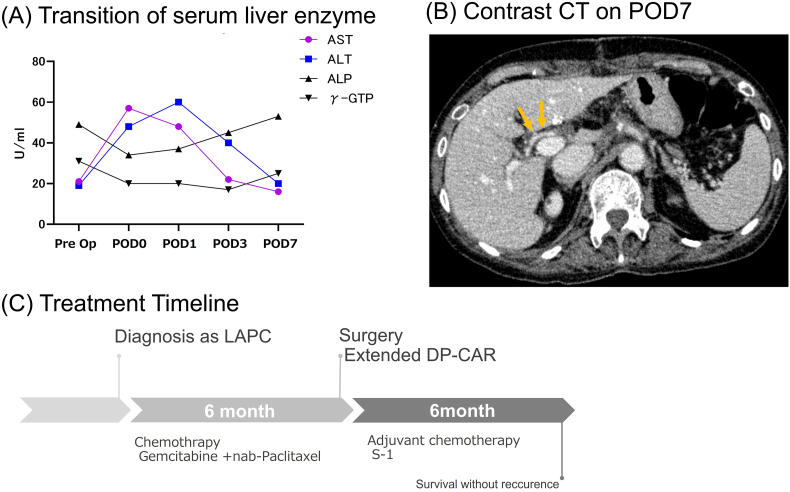
Postoperative course and the treatment timeline. (A) Postoperative transition of serum liver enzymes: serum levels of AST and ALT were within normal limits on POD7. (B) The right hepatic artery and whole liver parenchyma were well perfused on POD7. (C) The treatment timeline.

**Fig. 5:**
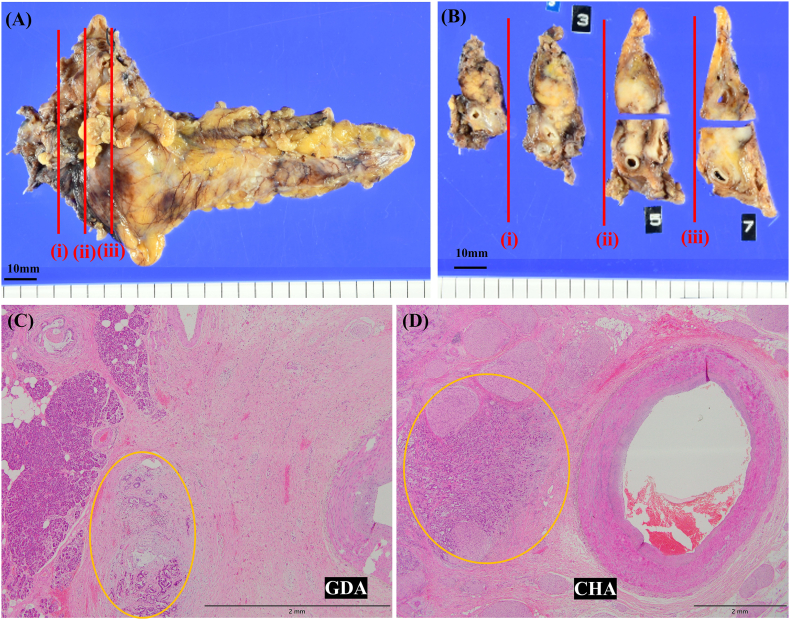
Specimen examination. Macroscopic findings showed the tumor surrounding the arteries. The maximum size of the tumor was 27 mm (A, B). Histologic examination of resected tissue samples (C, D). Hematoxylin and eosin (H & E) staining original magnification ×20 (C), ×40 (D). (C, D) Although there was no evidence of invasion of the arteries, periarterial fibrosis was evident (carcinoma cells: yellow circle). (For interpretation of the references to colour in this figure legend, the reader is referred to the web version of this article.)

We performed DP-CAR while preserving the LGA. The portal vein was resected with reconstruction. Combined resection of the GDA and the PHA, without revascularization, was conducted. D2 lymphadenectomy was also conducted (Fig. 3). The spleen was preserved. While the tissues around the root of the SpA and CHA were extremely stiff, those around the root of the LGA were not very stiff. The GDA and the PHA were noted to be involved in the tumor. We clamped both the celiac axis distal to the bifurcation of the LGA and the GDA. Doppler ultrasonography confirmed arterial flow to the liver during clamping. Therefore, we transected the CA and the GDA at the same point of clamping. The PHA was transected proximal to the bifurcation of the right gastric artery. Because the tumor had invaded the portal vein (PV)/superior mesenteric vein (SMV) and the splenic vein (SpV), we resected these vessels and reconstructed with end-to-end anastomosis of the PV and the SMV, end-to-side anastomosis of the left gastric vein and inferior mesenteric vein (IMV), and end-to-side anastomosis of the IMV and the PV. We performed cholecystectomy to avoid gallbladder ischemia. Serum liver enzymes were not elevated after the operation (Fig. 4A). Oral intake commenced two days after surgery. Although temporary elevations of hepatic enzymes were observed, normalization of aspartate aminotransferase (AST) and alanine transaminase (ALT) was obtained on postoperative day seven (Fig. 4A). Contrast abdominal CT at seven days after surgery showed good blood supply to the entire liver (Fig. 4B). The postoperative pancreatic fistula was grade BL (ISGPF 2016). The patient was discharged 34 days after surgery. Histopathological findings were as follows: invasive ductal carcinoma, maximum size was 27 mm, nodular type, all surgical margins were negative, no evidence of invasion to the artery, no lymph node metastasis, pathological stage II A, and histological assessment of therapeutic response was grade 1. Periarterial fibrosis was seen, indicating that chemotherapy killed the carcinoma cells around the arteries (Fig. 5). Adjuvant chemotherapy that consists of S-1100 mg/day from day 1 to day 28 was started a month after surgery. She had no evidence of the disease at her six-month follow-up visit (Fig. 4C).

## 3 Discussion

To our knowledge, this is the first English literature case of DP-CAR with combined resection of GDA (extended DP-CAR) by preservation of the replaced left hepatic artery. Our experience highlights the efficacy of preserving the replaced left hepatic artery and the importance of preoperative evaluation of arterial variations.

First, extended resection of the right surgical margin and combined resection of the GDA in DP-CAR was possible because of preservation of the replaced left hepatic artery. The existence probabilities of aberrant right and left hepatic arteries are 14 % and 13 %, respectively [4]. These are common variations. In a previous report, DP-CAR with combined resection of GDA was successfully performed in a case of pancreatic head and body cancer with replaced right hepatic artery by preserving the r-RHA [5]. This surgical method succeeded because the left lobe of the liver was supplied with enough blood from the r-RHA via the interlobar hepatic artery communications at the hepatic hilus. We wonder if it is similarly safe and feasible in opposite cases like the present case. Some studies have shown that intrahepatic arterial blood flow communicates via the interlobar hepatic artery at the hepatic hilus and the intrahepatic translator artery [6], [7], [8], [9], [10], [11]. Furthermore, collateral blood flow rapidly develops and compensates for the blood supply to the liver and bile duct if the hepatic artery is occluded [12].

Surgeons often encounter the r-RHA in pancreaticoduodenectomy. Usually, the r-RHA from the SMA runs dorsal of the pancreatic head and sometimes through the pancreatic head. When the r-RHA is invaded by a tumor, combined resection of revascularization of RHA is performed. There is a risk of lethal bleeding after pancreaticoduodenectomy in arterial reconstructions. Therefore, the efficacy and safety of combination therapy of preoperative coil embolization for the r-RHA and pancreaticoduodenectomy with combined resection of r-RHA without reconstruction was reported [13]. Furthermore, pancreaticoduodenectomy with combined r-RHA, without both preoperative embolization and reconstruction, was reported [14].

These procedures are also based on the theory mentioned above. This includes intrahepatic arterial blood flow communications and rapid development of collateral blood flow. Although there is concern regarding whether the replaced left liver artery can supply sufficient blood flow to the right lobe (that is bigger than the left lobe), the existence of collateral blood flow from the right inferior phrenic artery to the right lobe, as well as intrahepatic communication, is possibly associated with the feasibility of these surgical procedures including our case.

Second, it is important to perform a detailed preoperative evaluation for arterial variation. This sometimes allows for resection of unresectable pancreatic cancer, and can avoid arterial reconstruction that enhances mortality risk. Pancreatic cancer that has contact with the CA is classified as “locally advanced” and is often resected via DP-CAR [15]. If the tumor involves the GDA and the CA, it is unresectable, even by DP-CAR. However, a detailed evaluation of the arteries from the CA and SMA sometimes leads to radical resection for a basically unresectable pancreatic cancer. When deciding on resectability status preoperatively, it is necessary to pay attention to both the relationship between the tumor and the vessels and also the arterial variations. Three-dimensional CT simulation is useful for this.

As described above, replacement of the hepatic artery is not rare. When deciding the treatment strategy for locally advanced pancreatic cancer it is important to check the arterial variations. Surgeons must consider that one side of the hepatic artery can adequately supply the entire liver in almost all cases.

## 4 Conclusion

We successfully performed DP-CAR with combined resection of the GDA (extended DP-CAR) by preserving the r-LHA. Our case is the first English literature report of the r-LHA solely supplying the entire liver in extended DP-CAR. It is also important to note that although the resectability status for pancreatic cancer is decided according to the National Comprehensive Cancer Network guidelines, true resectability must be decided with close evaluations of both the tumor status and the vessel variations.

## Funding

This research did not receive any specific grant from funding agencies in the public, commercial, or not-for-profit sectors.

## Ethics approval and consent to participate

No ethical approval was required for the present case report. The patient consented to the publication of this case report.

## Consent for publication

Written informed consent was obtained from the patient's next of kin for publication of this case report and any accompanying images. Written informed consent was obtained from the patient for publication of this case report and accompanying images. A copy of the written consent is available for review by the Editor-in-Chief of this journal on request.

## Availability of data and materials

All relevant data are provided in the manuscript.

## Author contribution

Tetsunosuke Shimizu: Conceptualization, Methodology.

Koji Komeda: Data curation.

Atsushi Tomioka: Writing - Original draft preparation.

Nao Kawaguchi: Visualization, Investigation.

Mitsuhiro Asakuma: Writing - Reviewing and Editing.

Sang-Woong Lee: Supervision.

## Registration of research studies

Not yet.

## Guarantor

Atsushi Tomioka MD.PhD.

## Declaration of competing interest

There are no conflicts of interest to declare.
